# Editorial: Neural correlates of connected speech indices in acquired neurological disorders

**DOI:** 10.3389/fneur.2024.1550150

**Published:** 2025-01-21

**Authors:** Georgia Angelopoulou, Dimitrios Kasselimis, Georgios Velonakis, Dionysis Goutsos, Constantin Potagas

**Affiliations:** ^1^Neuropsychology and Language Disorders Unit, 1st Department of Neurology, Eginition Hospital, School of Medicine, National and Kapodistrian University of Athens, Athens, Greece; ^2^Department of Psychology, Panteion University of Social and Political Sciences, Athens, Greece; ^3^2nd Department of Radiology, General University Hospital “Attikon”, Medical School, National and Kapodistrian University of Athens, Athens, Greece; ^4^Department of Linguistics, School of Philosophy, National and Kapodistrian University of Athens, Athens, Greece

**Keywords:** connected speech, neuroanatomy, neurological disorders, oral speech production, narrative ability

Connected speech is considered a valuable methodological approach of increased ecological validity for the investigation of oral speech production, in both individuals with acquired neurological disorders and healthy speakers (1–3). Researchers from various scientific fields implement several measures to quantify narrative ability in order to extract conclusions about inner cognitive mechanisms while speaking. So far, limited evidence exists from the in-depth investigation of the structural and functional neuroanatomical correlates of discourse production in neurological disorders (4–6). The aim of our Research Topic is to advocate a better understanding of the neurobiology of interrupted narrative abilities in neurological populations with acquired language and further cognitive disorders. It incorporates evidence from distinct neurological populations, such as patients with right hemisphere stroke, patients with aphasia due to left hemisphere stroke and patients with epilepsy, highlighting the importance of a detailed connected speech analysis, along with in-depth investigation of their neural origins.

Meier et al. have investigated content unit production in a picture description task in patients with a history of left or right hemisphere stroke in relation to functional connectivity networks. Results revealed that left stroke patients produced significantly fewer content units per second compared to right stroke participants. No significant relationships were identified between content units' measures and connectivity of the major networks, such as dorsal attention, default mode, or language network for the right stroke group, based on neuroimaging analyses. However, increased number of content units per second was related with stronger left and weaker right frontotemporal connectivity. The authors concluded that such findings enhance the importance of language networking, as compared to other functional connectivity networks in oral speech production.

Angelopoulou et al. have implemented an integrative approach combining linguistic, neuropsychological and neuroanatomical data to investigate the frequency and duration of silent pauses, using two distinct narrative tasks (picture description and stroke story), in post-stroke aphasia. Their results revealed that patients pause more frequently and for longer durations during picture description, as compared to the stroke story task, while the opposite trend was observed for controls. Moreover, an interaction between semantic fluency and narrative task, as a function of pause location, seems to predict the duration of silent pauses. Finally, silent pauses duration can be predicted by spared tissue in frontal and parietal regions, as a function of the narrative task. The authors highlight the importance of silent pause metrics for the investigation of oral speech production using different narrative tasks.

Quique et al. have examined electrophysiological responses in a cohort of Spanish speakers with post-stroke aphasia while listening to continuous speech narration. More specifically, they estimated cortical tracking of the speech envelope (CTenv) in the delta, theta and alpha bands. Results indicated that only CTenv in delta and theta could significantly predict aphasia severity. Moreover, little association was found between rhythm perception and production with CTenv. Finally, CTenv in theta could predict sentence-level learning. The authors conclude that such preliminary findings indicate the importance of cortical activity in the theta and delta bands for speech processing in patients with acquired brain lesions.

Pracar et al. introduced an interesting case study of a patient suffering a fronto-insular ischemic stroke with apraxia of speech, investigating apraxic speech errors patterns and their underlying neural correlates. The authors examined the patient in three different moments, 1-, 3- and 12-months post-stroke. While during the first assessment, the patient indicated minor deficits in writing, auditory comprehension, and repetition, by the second assessment he no longer presented aphasia signs. However, the Motor Speech Evaluation revealed a moderate deficit that persisted after 12 months post-stroke. Neuroimaging data revealed a fronto-insular lesion including the superior precentral gyrus of the insula and portions of the inferior and middle frontal gyri and precentral gyrus, as well as the frontal aslant tract and arcuate fasciculus. The authors conclude that such pure cases of apraxia of speech may constitute a unique opportunity to investigate the neural origins and the significant role of specific cortical and subcortical areas in this motor speech disorder.

Finally, Floros et al. examined individuals with Mesial Temporal lobe Epilepsy and Juvenile myoclonic epilepsy, implementing an extensive neuropsychological and personality assessment. The results of the study have revealed that epilepsy type and lateralization seem to affect performance in executive function and pragmatics tasks. The authors indicate the importance of detailed investigation of cognitive abilities in different types of epilepsy, while they also highlight the importance of dissociating cognitive deficits from personality disorders in order to avoid possible misinterpretation.

To conclude, our Research Topic covers a wide range of thought-provoking issues related to the neurobiology of discourse production in various neurological populations, as researchers investigated distinct subcomponents of discourse production. This is of particular importance, since discourse production can be analyzed via various indices that have not yet been thoroughly studied. Studies included in the current issue contribute to the ongoing effort of the integrative investigation of narrative abilities, including neural correlates of its various parameters in populations with disrupted brain structure and function.
